# Advanced ossification of the carpal bones, and monkey wrench appearance of the femora, features suggestive of a propable mild form of desbeqious dysplasia: a case report and review of the literature

**DOI:** 10.1186/1757-1626-2-45

**Published:** 2009-01-13

**Authors:** Ali Al Kaissi, Christof Radler, Klaus Klaushofer, Franz Grill

**Affiliations:** 1 Ludwig-Boltzmann Institute of Osteology at the Hanusch Hospital of WGKK and AUVA Trauma Centre Meidling, 4th Medical Department, Hanusch Hospital, Vienna, Austria; 2 Orthopaedic Hospital of Speising, Paediatric Department, Vienna, Austria

## Abstract

**Intoduction:**

Advanced bone maturation is a radiographic feature that might be encountered in a number of different forms of skeletal dysplasias such as Desbuquois dyspalsia, Larsen syndrome, the Reunion Island form of Larsen syndrome, diastrophic dysplasia, acrodysostosis, Catel-Manzke syndrome, a variant of metatropic dysplasia and Maroteaux-lamy syndrome.

**Case presentation:**

We report on a 2-year- old boy from Slovakia was born to non-consanguineous parents. Prenatal and postnatal growth parameters were normal. Clubfoot and genu valgum were the most prominent orthopaedic abnormalities. Radiographic documentation showed bone age of 4 years and 8 months associated with the appearance of accessory ossification centers. Monkey wrench appearance of the proximal femora was a characteristic finding associated with significant vertebral changes.

**Conclusion:**

The major skeletal changes in our patient include advanced carpal ossification, monkey wrench appearance of the proximal femora associated with significant vertebral changes. No joint dislocations, no hitchhiker thumbs and or dysmorphic facial features were present. The normality of his growth, facial features, intelligence, and palate as well as the characteristic radiographic features were to certain extent in favour of a mild form of Desbuquois dysplasia. Additional laboratory findings allowed us to exclude other disorders with abnormal metabolic parameters such as mucopolysaccharoidosis.

## Introduction

Desbuquois dysplasia is a rare condition of autosomal recessive inheritance, characterised by marked short stature of prenatal onset, specific facial features, and joint laxity. In the classical and severe type of Desbuquois dysplasia "Swedish key appearance" of the proximal femur and advanced carpal and tarsal bone age are characteristic features. Birth length is generally less than -4 SD and final height reaches -10 SD of the mean. Hand changes include an extra ossification center distal to the second metacarpal, delta phalanx, bifid distal phalanx of the thumb, and phalangeal dislocations are additional skeletal characteristics. Other clinical manifestations comprise mid-facial hypoplasia, micrognathia with a high-arched palate, severe joint laxity, and occasional mental retardation [1-3].

The classical severe type of Desbuquois syndrome is reminiscent of Larsen syndrome, in that there is joint laxity with multiple dislocations. The eyes are prominent, the nasal bridge tends to be flat, and there can be marked micrognathia. Radiological changes are distinctive. There are supernumerary phalanges, characteristically situated between the metacarpal and proximal phalanx of the index finger, osteoporosis, a short narrow thorax, metaphyseal enlargement and platyspondyly [4]. Maroteaux et al [5], suggest that the prominence of the lesser trochanter at the hip as characteristic feature of the disorder. Ossification in the carpal centres may be advanced, whereas the epiphyses of the long bones can have retarded development.

## Case presentation

**Figure 1:**
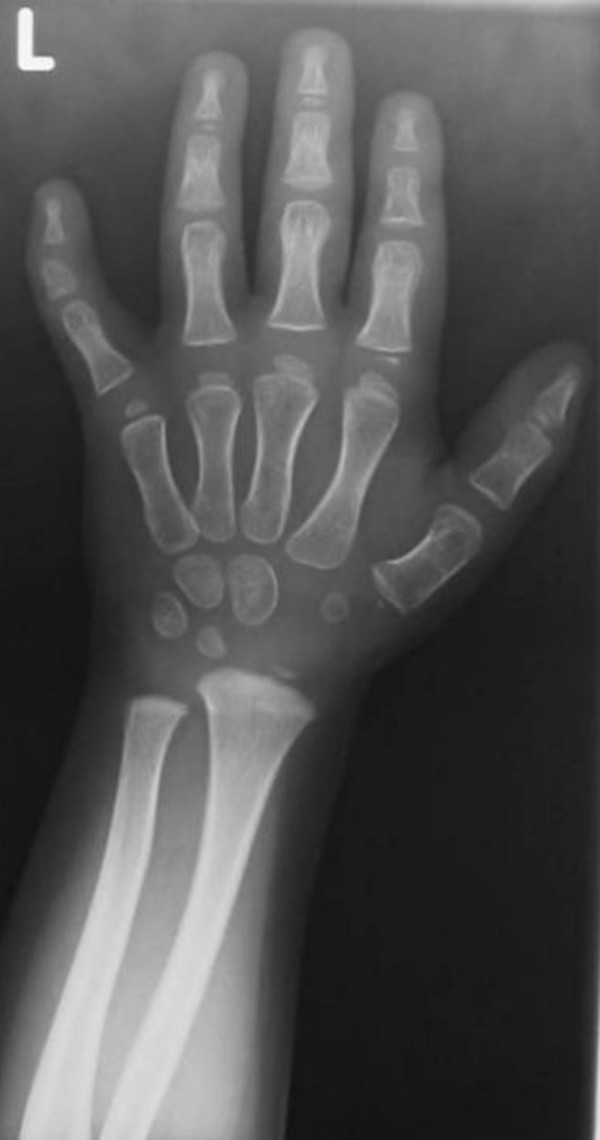
**Anteroposterior hand radiograph at the age of 2- years showed advanced carpal ossification centres equivalent to bone age of 4 years 8 months, supernumerary ossicles at the proximal portion of the first metacarpal and another at the proximal portion of the middle phalanx of the second metacarpal**. Delta like- middle phalanx of the fifth finger was evident.

**Figure 2:**
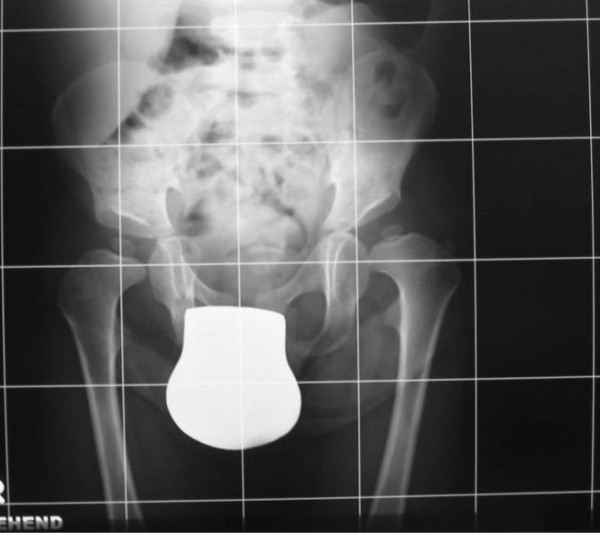
**Anteroposterior radiograph of the pelvis showed a mild monkey wrench appearance of the femora, elevated greater trochanters, coxa vara, rounded shaped dysplastic capital femoral epiphyses and a horizontally dysplastic acetabulae and a tongue-like projection of the lesser trochanters**.

**Figure 3:**
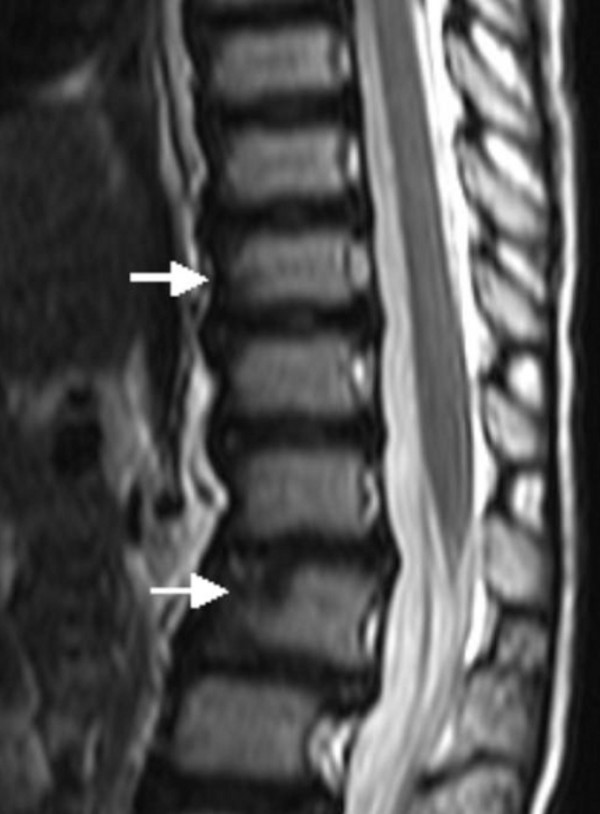
**Sagittal MRI imaging of the spine showed platyspondyly associated with anterior clefting of the 12 Th thoracic vertebra (arrow) and hook-like deformity of the 5 Th lumbar vertebra (fig 4)**.

A 2-year-old boy was referred to our department because of knock- knees (genu valgum) associated with mild stiffness over the ankle and knee joints. He was a product of uneventful gestation. At birth his growth parameters were around the 50 th percentile. At birth clubfoot was the predominating deformity, for which he was operated in Slovakia at the age of one year. The mother was a 31-year-old-gravida 1 abortus 0 married to a 33-year-old unrelated man. We did not have the opportunity to examine the parents. When examined at the age of 2 years, he had normal craniofacial contour, normal facial features and normal growth (fig 1). No apparent deformation of the hands, except for clinodactyly of the 5th finger. His subsequent course of development has been of normal limits. Hearing, vision and intelligence were normal. Musculoskeletal showed mild stiffness over the wrists, knees and ankles were present. No specific skin stigmata have been encountered. On the bases of skeletal survey, anteroposterior hand radiograph at the age of 2- years showed advanced carpal ossification centres equivalent to bone age of 4 years 8 months, supernumerary ossicles at the proximal portion of the first metacarpal and another at the proximal portion of the middle phalanx of the second metacarpal. Delta like- middle phalanx of the fifth finger was evident (fig 1). The anteroposterior radiograph of the pelvis showed a mild monkey wrench appearance of the femora, coxa vara, elevated greater trochanters, rounded shaped dysplastic capital femoral epiphyses and a horizontally dysplastic acetabulae and a tongue-like projection of the lesser trochanters (fig 2). Sagittal MRI imaging of the spine showed platyspondyly associated with anterior clefting of the 12 th thoracic vertebra (arrow) and hook-like deformity of the 5 th lumbar vertebra (fig 3). Ophthalmological, audiological and neurological examinations were normal, as were blood biochemistry and screening for mucopolysaccharoidosis were normal.

Laboratory tests aimed to assess thyroid hormones, calcium, phosphorus, and alkaline phosphatase were normal. Screening for mucopolysaccharoidosis gave normal results. No specific molecular investigations have been done for this patient.

## Discussion

Desbuquois dysplasia is a well-elucidated osteochondrodysplastic disorder inherited as an autosomal recessive trait. The clinical features are characterised by severe micromelic short stature, severe joint laxity, flat mid-face, micrognathia with cleft palate, and occasional mental retardation. In severe cases joint hyperlaxity with joint dislocation is a major orthopaedic abnormality. The major radiographic criteria include five consistent features, namely monkey-wrench/Swedish-key appearance of the proximal femora, flat acetabular roof, elevated greater trochanter, precocious carpal and tarsal ossification and proximal fibular overgrowth [1-5]. Nishimura et al., [6] reported 3 cases with a possible mild form of Desbuquois dysplasia. There was generalised osteopenia, broad proximal femora and prominent lesser trochanters (a monkey wrench appearance of the proximal femora). There was also mild epiphyseal flattening of the long bones. Growth failure was postnatal and there were minor joint problems, including exaggeration of bowlegs as a toddler, mild hand joint laxity, metatarsus adductus, flat feet, hip subluxation, and mild joint restriction. Bone age was advanced, particularly in the hand. There was mild platyspondyly in mid-childhood with somewhat biconvex vertebral bodies. Al Kaissi et al [7] reported a Tunisian family with a condition resembling Desbuquois dysplasia. It differed from the classical condition in that the face was normal and that joint hyperlaxity, although initially present (with dislocations), did not persist (it developed into painful stiffness) and the hands showed small, round intermetacarpal seasamoid bones.

Diastrophic dysplasia is characterised by the hallmark of severe short limb dysplasia noted at birth by short limbs, especially rhizomelic shortening, severe talipes, hitch-hiker thumbs, a cleft palate in many, a characteristic swelling of the pinnae, which assumes the character of a cauliflower ear, and occasional dislocations of joints. Respiratory problems, due to a narrow chest and micrognathia, can be a cause of early death. Progressive abnormal curvature of the spine, and unusually positioned thumbs (abducted-hitchhiker thumbs) are characteristic features. Hall [8] described extreme variability within sibships in which 3 sibs were diagnosed with diastrophic dysplasia. He commented that the phenotype might be sufficiently mild in some instances as to render the diagnosis uncertain.

Advanced carpal ossification is a feature that might be encountered in Larsen syndrome, the Reunion Island form of Larsen syndrome, acrodysostosis, and in rare variant of metatropic dysplasia [5]. Nishimura et al [9] described advanced carpal skeletal age and subluxation of the radial heads in children with a variant of metatropic dysplasia. The overall clinico-radiographic features were incompatible with our present patient.

## Conclusion

Distinctive radiographic futures in our patient include precocious carpal bone ossification associated with supernumerary ossicles at the proximal portion of the first metacarpal and another ossicle at the proximal portion of the middle phalanx of the second metacarpal and Delta like- middle phalanx of the fifth finger. The monkey wrench appearance of the proximal femur, a horizontally dysplastic acetabulae associated with significant spine changes are features consistent with Desbuquois dysplasia. Neither facial dysmorphic features nor joint dislocations or growth deficiency were present in our patient. We suggest that our patient might manifest either a mild variant of Desbuquois dysplasia or a Desbuquois-like disorder.

## Abbreviations

SD: Standard deviation.

## Consent

Written informed consent was obtained from the parents for the purpose of publication of the manuscript and figures of their child. A copy of the written consent is available for review by the editor-in-Chief of this journal.

## Competing interests

The authors declare that they have no competing interests.

## Authors' contributions

All of the authors were involved in the clinico-radiographic assessment and finalising the paper. All authors have red and approved the final version of the paper.
